# Precursor T-cell acute lymphoblastic leukemia presenting with bone marrow necrosis: a case report

**DOI:** 10.1186/1752-1947-6-349

**Published:** 2012-10-11

**Authors:** Najmaddin SH Khoshnaw, Hisham A Al-Rawi, Beston F Nore

**Affiliations:** 1 Department of Hematology, Hiwa Hematology-Oncology Center, Kurdistan Regional Government, Sulaimaniyah, Iraq; 2 Department of Hematopathology, University of Sulaimani, Kurdistan Regional Government, Sulaimaniyah, Iraq; 3 Department of Biochemistry, Faculty of Medical Sciences, School of Medicine, University of Sulaimani, Kurdistan Regional Government, Sulaimaniyah, Iraq

**Keywords:** Bone marrow necrosis, Acute lymphoblastic leukemia, Bone marrow aspiration

## Abstract

**Introduction:**

Bone marrow necrosis is a clinicopathological condition diagnosed most often at postmortem examination, but it is also seen during the course of malignancy and is not always associated with a poor prognosis. The morphological features of bone marrow necrosis are disruption of the normal marrow architecture and necrosis of myeloid tissue and medullary stroma. Non-malignant conditions associated with bone marrow necrosis are sickle cell anemia, infections, drugs (sulfasalazine, interferon α, all-trans retinoic acid, granulocyte colony-stimulating factor and fludarabine), disseminated intravascular coagulation, antiphospholipid antibody syndrome and acute graft versus host diseases. The malignant causes are leukemia, lymphoma and metastatic carcinomas. Herein we report the case of a patient with precursor T-cell acute lymphoblastic leukemia and bone marrow necrosis at initial presentation.

**Case presentation:**

A 10-year-old Kurdish boy was presented with generalized bone pain and fever of 1 month’s duration which was associated with sweating, easy fatigability, nose bleeding, breathlessness and severe weight loss. On examination, we observed pallor, tachypnea, tachycardia, low blood pressure, fever, petechial hemorrhage, ecchymoses, tortuous dilated veins over the chest and upper part of abdomen, multiple small cervical lymph node enlargements, mildly enlarged spleen, palpable liver and gross abdominal distention. Blood analysis revealed pancytopenia and elevated lactate dehydrogenase and erythrocyte sedimentation rate. Imaging results showed mediastinal widening on a planar chest X-ray and diffuse focal infiltration of the axial bone marrow on magnetic resonance imaging of the lumbosacral vertebrae. Bone marrow aspiration and biopsy examination showed extensive bone marrow necrosis. Immunophenotyping analysis of the bone marrow biopsy confirmed T-cell acute lymphoblastic leukemia, as CD3 and terminal deoxynucleotidyl transferase markers were positive and CD10, CD20 and CD79a markers were negative.

**Conclusion:**

The aggressive initial clinical presentation of our patient with huge mediastinal widening, development of superior vein cava syndrome and extensive bone marrow necrosis as initial signs made the diagnosis of the case difficult. The necrotic hematopoietic cells gave inconclusive results on the initial immunohistochemistry tests. The prognosis of bone marrow necrosis is better secondary to acute lymphoblastic leukemia in the pediatric age group compared with adults and those with underlying solid tumors. Despite the aggressive behavior at initial presentation, the patient responded to chemotherapy and necrosis disappeared at day 28 after the start of the therapeutic regimen.

## Introduction

Bone marrow necrosis (BMN) is a relatively uncommon clinical and pathological entity [1]. It is often overlooked in the antemortem period [2]. BMN is poorly understood and frequently an unrecognized finding in routine bone marrow biopsies. However, the pathologic entity related to necrosis of myeloid tissue and medullary stroma is well-described in the literature [3,4]. Actually, BMN is noted more often in trephine sections than in aspirates [5]. The bone marrow biopsy specimen shows an increase in eosinophilic granular stroma along with ghosts of many dead hematopoietic or tumor cells [1]. BMN changes have been found to be widespread and to occur immediately, before or during chemotherapy [6]. Thus, BMN is diagnosed most often in autopsied than living patients [7-11]. General features of BMN are bone pain, fever, anemia, thrombocytopenia, leukoerythroblastic picture, elevated lactate dehydrogenase, elevated alkaline phosphatase, hypercalcemia and jaundice [1,6,12-15].

The etiology of BMN is malignant and non-malignant conditions. The non-malignant causes are acute graft versus host disease after allogeneic bone marrow transplantation (BMT), megaloblastic/sideroblastic anemia [3,8], severe disseminated intravascular coagulation [16], antiphospholipid antibody syndrome [3,17], severe infections, especially bacterial infection (with hypovolemia and septic shock) [3,17] and bone marrow transplant (BMT) [3,14].

The malignant causes are first hematological as acute lymphoblastic leukemia (ALL) [3,8], acute myelogenous leukemia, chronic myelogenous leukemia, myeloma, Hodgkin’s disease [3,18], non-Hodgkin’s lymphoma (NHL) [19,20] and high-grade B-cell lymphoproliferative disease (central nervous system involvement and hypercalcemia) [3,7]. Fulminant BMN that occurred after infusion of fluduabine monophosphate in a patient with recurrent low-grade NHL has been reported previously [20]. Experience with induction therapy for acute promyelocytic leukemia, both with and without all-*trans* retinoic acid therapy, suggests that the addition of hydroxyurea is critical to the development of BMN caused by massive cell lysis and death [21]. Also, it has been shown to occur during treatment with interferon α in some patients because of production of tumor necrosis factor (TNF) α by mononuclear cells [22] and, secondarily, non-hematologically in various types of carcinoma such as neuroblastoma.

BMN is best defined as necrosis of myeloid tissue and medullary stroma in large areas of the hematopoietic bone marrow [5]. Trephine sections show that, in necrotic areas, the marrow architecture is destroyed and the supporting connective tissue is absent [3]. The necrosis is characterized by a disruption of the normal bone marrow architecture and a considerable loss of fat spaces. This contrasts with the findings in aplastic anemia, in which there is only a loss of myeloid tissue and no destruction of the reticular structure. Usually, there is no destruction of the spicular architecture as is seen in aseptic necrosis [13]. The absence of BMN in the setting of all cells scattered can be seen in a background of amorphous eosinophilic staining material; in several cases, increased fibrosis has been observed [1,13]. Scintigraphy was typically used in the past to evaluate the extent of BMN, but magnetic resonance imaging (MRI) is currently preferred [7]. The pathophysiology of BMN is not well-understood. It has been shown that necrosis is mediated by cytotoxic T cells [3] or the release of either toxins or soluble mediators by malignant cells [20]. Cytokines such as TNF may induce expression of leukocyte adhesion receptors on endothelial cells [4]. Granulocyte activation with generation and release of superoxide has a prothrombotic effect on endothelial cells [4].

The prognosis of BMN depends on the patient’s age and the underlying pathology [23]. Children with a BMN appearance together with ALL have been shown to enter remission following standard treatment that results in complete marrow healing [23,24], but in mortality in others [24,25]. It is known that the prognosis of adults with BMN secondary to neoplastic disease is extremely poor; however, this prospect alone may not be applied to children with ALL [23,24,26]. We conclude that antemortem diagnosis of BMN is technically difficult, but as it is not always associated with a fatal prognosis, early diagnosis and vigorous supportive therapy should be attempted [25,27,28].

## Case presentation

**Figure 1:**
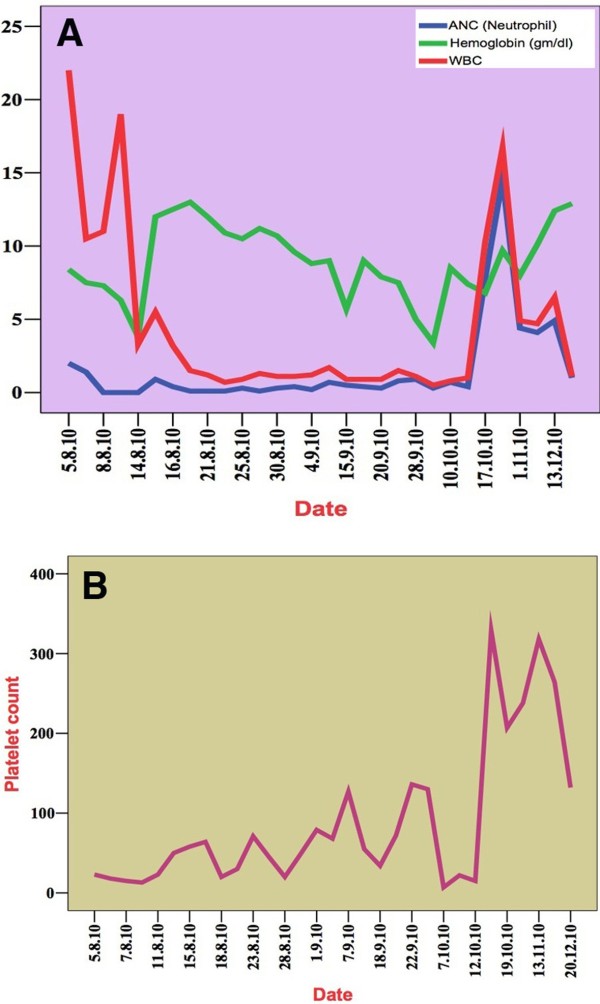
**Clinical course of the patient regarding peripheral cell counts, including absolute neutrophil count, hemoglobin, white blood cells (A) and platelets (B). **The white blood cell count (red areas in **A**) was high on the first presentation, then decreased to its lowest level during the first month of induction therapy. The hemoglobin (green area in **A**) curve was elevated after induction because of the destruction of malignant cells by chemotherapy. The absolute neutrophil count (blue) was low from the beginning through the induction treatment period. All three parameters were markedly elevated after completion of the induction treatment phase. The platelets curve (**B**) indicates severe thrombocytopenia with a subsequent increase in platelets until reaching 300,000 cells at 40 days after induction treatment. ANC, Absolute neutrophil count; WBC, White blood cells.

**Figure 2:**
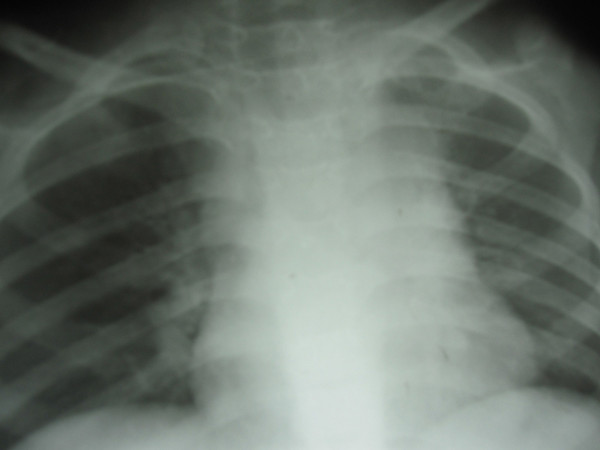
Chest X-ray showing mediastinal dilation at first presentation.

**Figure 3:**
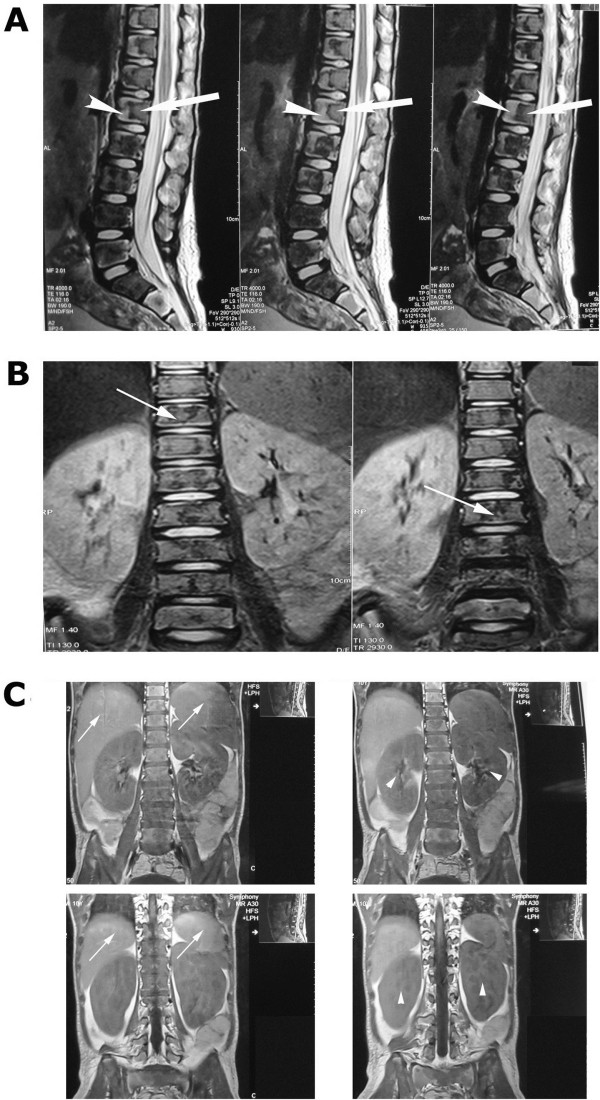
**Magnetic resonance imaging of the patient. **(**A**) A side view showing partial loss of normal hydration on hypointense T2-weighted signal intensity of the dorsal and lumbar intervertebral discs, as well as diffuse focal infiltration of the axial bone marrow of the lower dorsal and lumbar vertebrae, causing altered hypointense T1-weighted signal intensity (arrows), in homogeneous hyperintense T2-weighted signal changes (arrowheads). (**B**) Anteroposterior view. (**C**) Incidental hepatosplenomegaly (arrows) with bilateral renal enlargement (arrowheads).

**Figure 4:**
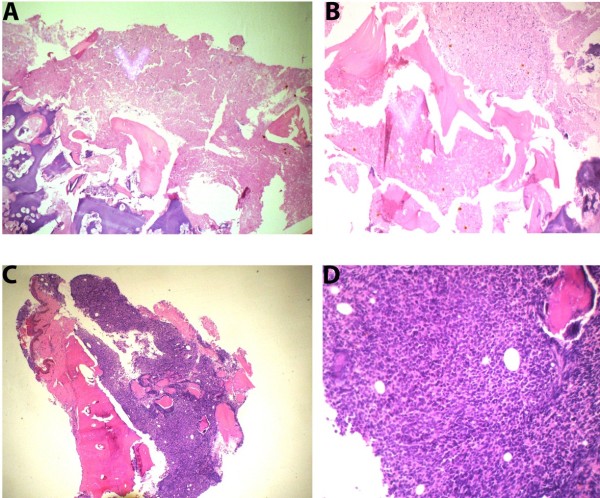
**Bone marrow aspiration biopsy (hematoxylin and eosin stain, ×10 magnification) at diagnosis (A) and (B). **These images show cells losing normal staining characteristics, as well as coagulative necrosis of marrow elements and bony trabeculae. The red arrow shows the area of necrosis. Necrosis of the myeloid tissue on a background of amorphous eosinophilic material is shown. (**C**) and (**D**) Images obtained at the time of relapse show infiltration of the bone marrow by malignant mononuclear cells.

A 10-year-old Kurdish boy presented with bone pain and fever associated with night sweats, shortness of breath, weight loss (5kg/month), purple purpuric spots over the skin and bleeding from the nose. The patient’s history dated back to 1 month before admission. On examination, we observed pallor, cachexia, dyspnea, fever, tachycardia, tachypnea, multiple petechiae and ecchymoses all over the skin, dilated tortuous veins over the chest, pulse rate 120bpm, respiratory rate 23cycles/minute, temperature 39°C and blood pressure 90/45mmHg. Moreover, we found multiple small cervical lymphadenopathies and mild splenomegaly 3cm below the left costal margin. The boy’s liver was tender 7cm below the right costal margin, and he had gross abdominal distention. The initial blood counts were hemoglobin 64g/L, white blood cell count 34 × 10^9^/L, platelets 25 × 10^9^/L and blasts 38% (Figure 1A). The blasts were homogeneous with a high nuclear to cytoplasmic ratio, inconspicuous nucleoli and open chromatin, and some of the blasts were vacuolated. Platelets were markedly reduced (Figure 1B). There was mediastinal widening visualized on a chest X-ray (Figure 2). MRI showed lumbosacral vertebrae with diffuse infiltration of the axial bone marrow of the lower dorsal and lumbar vertebrae, suggestive of bony metastases predominantly osteolytic in nature (Figure 3A– 3C). Bone marrow aspiration (BMA) showed no fragments but few areas of necrosis. Bone marrow biopsy showed marked BMN (Figure 4A and 4B). The first immunophenotyping was not conclusive, but the second was positive for CD3 and terminal deoxynucleotidyl transferase and negative for CD20, CD79a and CD10.

After admission, the patient’s condition deteriorated and features of frank superior vena cava syndrome developed. The patient was treated with chemotherapy according to the ALL protocol, and complete remission was achieved on day 28. At the 24th week of chemotherapy, his condition relapsed on treatment. He returned to our hospital with fever, chest infection and 60% blasts observed on peripheral blood film, and the complete blood count revealed severe pancytopenia. The BMA was a dry tap, but the biopsy showed hypercellular marrow with extensive infiltration by mononuclear cells and disappearance of necrosis (Figure 4C and 4D). Treatment was reinitiated according to the ALL protocol (bone marrow relapsed protocol), after which he developed severe mucositis, uncontrolled septicemia and electrolyte imbalance. Eventually, that led to death.

The BMA at the time of diagnosis was diluted, and the slides appeared to show artefact with a few necrotic cells. The biopsy was a 1.6cm piece of tissue that consisted of a fragment of trabecular bone showing marked BMN, as shown in the image in Figure 4A (before treatment). The result of immunohistochemistry was not interpretable for the first specimen, but for the second the diagnosis was definitive as precursor T-cell ALL.

During admission, we gave the patient intravenous fluid 3000ml/m^2^/day, allopurinol tablets 100mg/m^2^/dose and antibiotics. The patient’s condition subsequently deteriorated, and he developed progressive dyspnea, chest tightness, abdominal distention and fever. The patient was near to developing frank features of superior vena cava syndrome, but later he developed bilateral lower-limb weakness. The straight leg raising test was observed to be positive bilaterally. MRI of the dorsolumbosacral spine showed diffuse focal infiltration of the axial bone marrow of the lower dorsal and lumbar vertebrae causing altered hypointense T1-weighted signal intensity (Figure 3A and 3B). The image was suggestive of bony metastasis that was predominantly osteolytic in nature (Figure 3A and 3B). Incidental hepatosplenomegaly and bilateral renal enlargement were also observed (Figure 3C), but there was no pressure on the spinal cord.

We started dexamethasone intravenous infusion at 6mg/m^2^. Seven days after the patient’s admission, we started induction therapy with vincristine 1.5mg/m^2^ intravenous bolus on days 7, 14, 21 and 28. Dexamethasone 6mg/m^2^ was administered daily, and daunorubicin 25mg/m^2^ was given on the days 1 and 7.

Upon starting induction, the patient developed attacks of tonic-clonic contractions. We found computed tomography of the brain without contrast to be negative. Electrolyte measurements showed severe hypocalcemia, and we induced correction, which stabilized the convulsions. On day 28, BMA indicated a few fragments and megakaryocytes were seen. Erythroid and myeloid series were present with all stages of maturation. The data also indicated predominant neutrophils and histiocytes, but the cellular elements could not confirm an excess of blast cells. The bone marrow biopsy report showed 95% cellularity, which was composed predominantly of early-stage granulocytes and normal maturation stages of hematopoietic cells. The blasts constituted about 2% of total marrow nucleated cells. The myeloid to erythroid cell ratio was 8:1.

After a 4-week induction period, complete remission was observed and we continued giving the early consolidation chemotherapy. Unfortunately, at the 24th week of treatment, the patient returned with fever, chest infection, neutropenia and thrombocytopenia, and we found the presence of a few blasts in the peripheral blood film. The patient was not responding to supportive treatment that included antibiotics and antipyretics. The follow-up analysis of BMA showed excessive bone marrow infiltration by mononuclear cells with multiple inconspicuous nucleoli. Both erythroid and megakaryocytic precursors were suppressed in BMA, whereas in relapsed biopsy no necrosis was observed (Figure 4C and 4D).

On the reinduction therapy date, we followed a bone marrow relapsed protocol. On the 14th day of treatment reinduction, the patient developed severe anemia, thrombocytopenia and neutropenia. In addition, he developed grade IV mucositis with hypokalemia. The patient could not tolerate the complications, and he developed septicemia followed by sepsis. The patient’s death was an inevitable outcome. The overall survival period was 26 weeks after first diagnosis.

## Discussion

BMN is a rare clinicopathological condition that is rarely diagnosed at antemortem examination. In our case, BMN was the initial presentation of precursor T-cell ALL in a pediatric patient. However, the overall aim of presenting this case is to state that, after administering conventional chemotherapy, we found responses for even severe necrosis in an aggressive type of leukemia. Despite limited resources and facilities, our treatment with a lower quality of drugs elevated the patient’s survival time for more than 26 weeks. A definitive diagnosis of BMN is difficult; therefore, with two trials of BMA, the biopsy analysis still was not sufficient. We knew that BMN as the initial presentation preceding an ALL diagnosis has been described to have a bad prognosis. With early diagnosis and supportive treatment, the outcome is fair, such as in our present case, in which the necrosis had disappeared on the last BMN examinations, which indicated a positive response to treatment.

## Conclusions

BMN is usually diagnosed at postmortem examination. However, precursor T-cell acute lymphoblastic leukemia may present with BMN initially.

## Consent

Written informed consent was obtained from the patient’s parents for publication of this case report and any accompanying images. A copy of the written consent is available for review by the Editor-in-Chief of this journal.

## Competing interests

The authors declare that they have no competing financial interests.

## Authors’ contributions

NSHK performed most of the clinical diagnosis, treatment and evaluation, in addition to collecting all data and presenting the case. HAAR participated in most of the pathological images. BFN designed, restructured and presented most parts of the manuscript, including the references, and also contributed to the writing and editing of the manuscript. All authors read and approved the final manuscript.
